# Increased Asymmetric and Multi-Daughter Cell Division in Mechanically Confined Microenvironments

**DOI:** 10.1371/journal.pone.0038986

**Published:** 2012-06-25

**Authors:** Henry Tat Kwong Tse, Westbrook McConnell Weaver, Dino Di Carlo

**Affiliations:** 1 Department of Bioengineering, University of California Los Angeles, Los Angeles, California, United States of America; 2 California NanoSystems Institute, Los Angeles, California, United States of America; Emory University/Georgia Insititute of Technology, United States of America

## Abstract

As the microenvironment of a cell changes, associated mechanical cues may lead to changes in biochemical signaling and inherently mechanical processes such as mitosis. Here we explore the effects of confined mechanical environments on cellular responses during mitosis. Previously, effects of mechanical confinement have been difficult to optically observe in three-dimensional and *in vivo* systems. To address this challenge, we present a novel microfluidic perfusion culture system that allows controllable variation in the level of confinement in a single axis allowing observation of cell growth and division at the single-cell level. The device is capable of creating precise confinement conditions in the vertical direction varying from high (3 µm) to low (7 µm) confinement while also varying the substrate stiffness (E = 130 kPa and 1 MPa). The Human cervical carcinoma (HeLa) model with a known 3N+ karyotype was used for this study. For this cell line, we observe that mechanically confined cell cycles resulted in stressed cell divisions: (i) delayed mitosis, (ii) multi- daughter mitosis events (from 3 up to 5 daughter cells), (iii) unevenly sized daughter cells, and (iv) induction of cell death. In the highest confined conditions, the frequency of divisions producing more than two progeny was increased an astounding 50-fold from unconfined environments, representing about one half of all successful mitotic events. Notably, the majority of daughter cells resulting from multipolar divisions were viable after cytokinesis and, perhaps suggesting another regulatory checkpoint in the cell cycle, were in some cases observed to re-fuse with neighboring cells post-cytokinesis. The higher instances of abnormal mitosis that we report in confined mechanically stiff spaces, may lead to increased rates of abnormal, viable, cells in the population. This work provides support to a hypothesis that environmental mechanical cues influences structural mechanisms of mitosis such as geometric orientation of the mitotic plane or planes.

## Introduction

An immense amount of past and current research is dedicated to understanding the control systems that govern the very complex network of chemical reactions that dictate cell biology. Of these cellular control systems, perhaps the most extensively studied and complex is the cell cycle regulatory system. Cell cycle regulation controls the progression of the life cycle of a cell, the growth of tissues, and is ultimately a significant contributor to the physiological homeostasis of complex multicellular organisms. However, recent research have also shown that non-conventional mitosis events contribute to natural genetic variation [1], as well as tumor progression[2]–[5].

Over a half century of research, sparked by Howard and Pelc’s observation that radio-labeled phosphorous incorporates differentially into cells not undergoing mitosis [6], has resulted in an increasingly complex understanding of cell cycle regulation. Regulation of the cycle depends on the constant production and degradation of proteins, and the activation or deactivation of the complexes responsible for targeting these proteins for degradation via ubiquitination. Environmental cues such as soluble factors have long been implicated in the cell cycle control system, however the past decade has given a new perspective on mechanical cues involved in cell biology. In order to gain a more complete understanding of cell biology and the cell cycle, considering both soluble and mechanical cues will be necessary.

Mechanosensing is an important component of the physiology of the cell, as well as tissue homeostasis. Direct linkages between the extracellular matrix (ECM) and the intracellular environment allow external mechanical cues to alter the cellular state[7]–[9]. Conversely, these same linkages enable the cell to transmit forces extracellularly, altering the mechanical micro-environment itself [10]. Tipping this mechanical balance can result in cellular differentiation [11], morphology [12] and motility changes [13], as well as alterations in cell cycle control [14].

Mitosis is a highly regulated stage of the cell cycle, both biochemically and, more increasingly suspected, mechanically. The overall spherical shape that cells adopt during this phase and the internal organization of the cytoskeleton are directly implicated in influencing the progression through mitosis [15]. The Spindle Assembly Checkpoint (SAC) has been identified as the major checkpoint responsible for ensuring correct chromosomal alignment during metaphase [16]. The SAC requires specific mechanical cues to proceed through mitosis, including microtubule-kineticore attachments as well as sufficient tension in microtubules themselves [17], the satisfaction of which results ultimately in cytokinesis and mitotic exit [18].

The cell division axis is also dependent on the orientation of ECM near the dividing cell and this effect requires an intact actin cytoskeleton [19]. This link between the ECM orientation, cytoskeleton, and condensed DNA is further supported by the co-localization of cytoskeletal binding proteins and the spindle apparatus during mitosis [20]. Centrosome number and polarity has been shown to depend not only on an intact cytoskeleton [21], but also on the phosphorylation state of focal adhesion kinase (FAK), further implicating a delicate force balance during mitosis [22].

**Figure 1:**
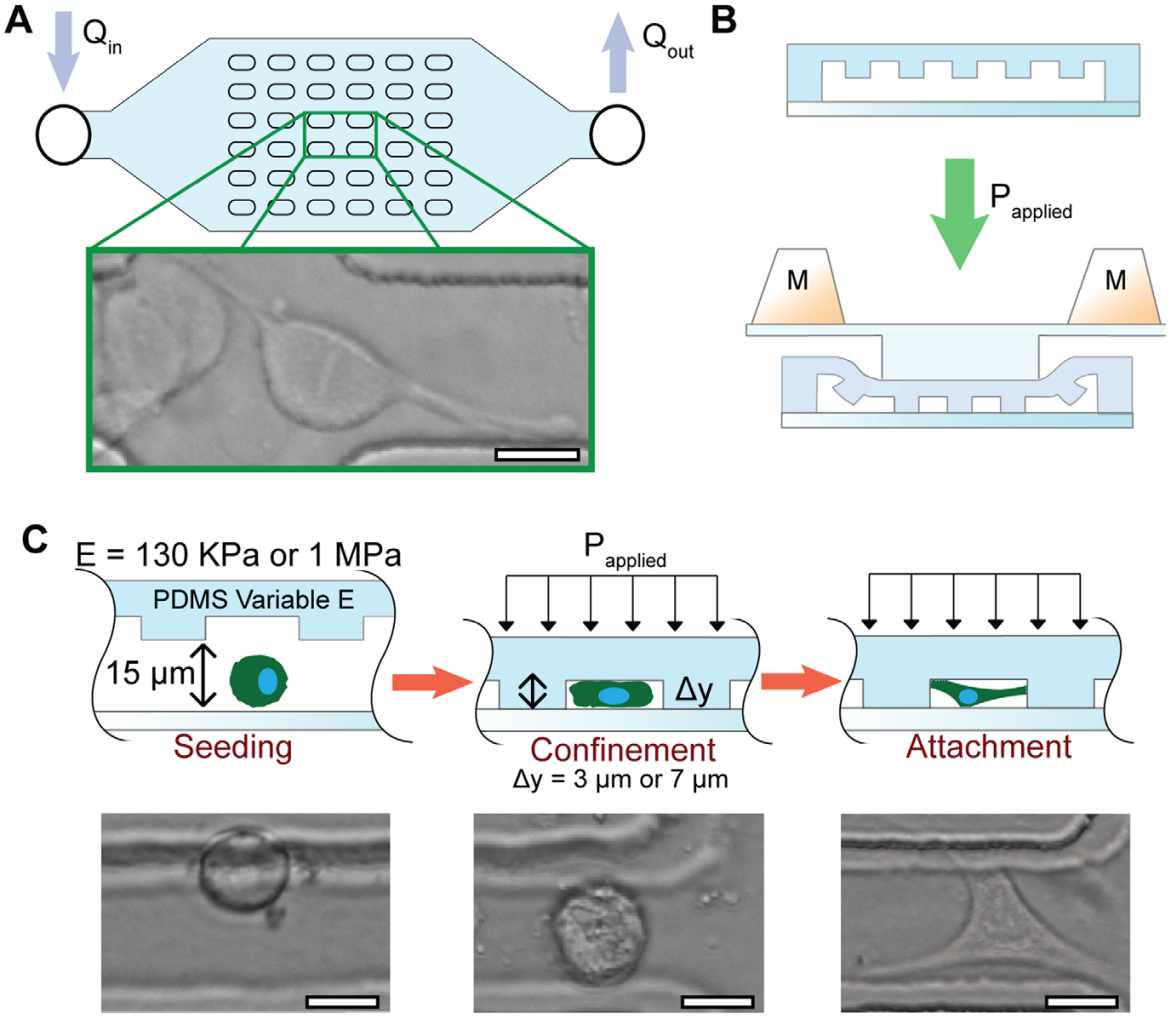
Microfluidic cell confinement device. (a) Device schematic with inset of confinement assay. The posts are 20×80 µm spaced equally 40 µm apart. (b) In the unconfined state, the posts are raised from the glass substrate (top), upon applying pressure the confinement chamber is compressed such that the posts are in contact with the glass substrate. (c) Seeding cells in the confinement chamber (left), when the posts are lowered the cell is confined to 3 or 7 µm height and is squeezed out from the post area (middle). Cells spread and attach in the confined volume (right).

We propose that aberrant mitotic outcomes, possibly due to altered cytoskeletal mechanics during mitosis, can be directed by an altered mechanical microenvironment. To explore this hypothesis, we have developed a novel microfluidic platform to confine a population of proliferating HeLa cells. This novel culture platform allows for both alterations in the geometry of the microenvironment, specifically the space in which the cell is allowed to grow and divide, as well as the elasticity of the substrate on which the cell is dividing. Figure 1 illustrates the device, with an example of a mitotic cell directly interacting with the elastic substrate (Fig. 1a). By using a microfluidic device to compress the cells, we minimize cell death due to lack of nutrients, as media is constantly perfused through the compression chamber. The device also allows for facile imaging of cells, as they are in a single focal plane.

## Results and Discussion

**Figure 2:**
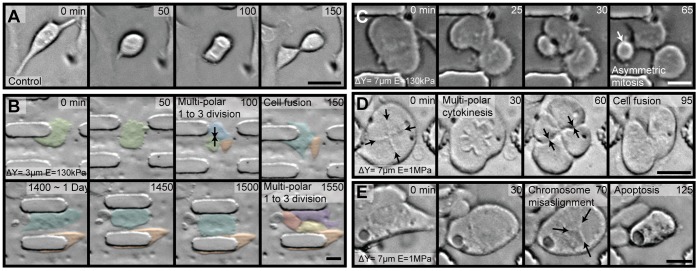
Abnormal cell divisions under mechanical confinement. (a) In an unconfined device control, mitosis occurs within 150 minutes. (b) A confinement condition (Δy = 3 µm, *E* = 130 kPa) showing sequential tri-daughter divisions with viable daughter cells. The first tri-daughter division is seen at 100 minutes, with cell fusion labeled by black arrows. The second tri-polar division is seen at 1550 minutes along with the viable 2^nd^ daughter cell labeled in orange. (c) A confinement condition (Δy = 7 µm, *E* = 130 kPa) showing an asymmetric multi-daughter division of cytoplasm after mitosis (cell with arrow receives disproportionate cytoplasmic volume). (d) A confinement condition (Δy = 7 µm, *E* = 1 MPa) showing a tetra-daughter division with a cross geometry chromosome alignment (marked by black arrows at 0 minutes) and cell fusion at 60 minutes (labeled by black arrows). (e) A confinement condition (Δy = 7 µm, *E* = 1 MPa) showing a triangular chromosome arrangement (marked by black arrows) leading to cell death. All scale bars are 20 µm.

Cells in confined 3D cultures exhibit drastic changes in size, shape, and symmetry of daughter cells when compared to unconfined 2D cultures. The height confinement also readily allowed visualization of condensed chromosomes at the mitotic plane. Within a 600 µm^2^ field-of-view cells are observed under time lapse bright-field microscopy for mitosis events (Video S1). In the unconfined device control, with posts in the up position (Fig. 2a), cells attain a spherical geometry during mitosis and complete the mitosis process within 140 minutes. However, in confined conditions, tri-daughter cytokinesis (Fig. 2b & 2c, Video S2 & S3), daughter cells with drastically different sizes (Fig. 2c, Video S3), tetra-daughter cytokinesis (Fig. 2d, Video S4), and mitosis resulting in cell death (Fig. 2e, Video S5) are commonly observed events that increase in frequency with increasing compression (decreasing Δy) and increasing stiffness (*E*). Since multi-polar divisions have previously been classified as divisions containing multiple centrosomes (N>2) during mitosis, to avoid confusion we refer to multi-daughter divisions as observable division events resulting in more than 2 daughter cells (including observations of tri-daughter through penta-daughter cytokinesis).

Multi-daughter divisions induced by confinement lead to viable daughter cells with increased chances of chromosomal abnormalities. Interestingly, in figure 2b sequential tri-daughter divisions in consecutive cell cycles are observed for the high confinement, low stiffness condition (Δy = 3 µm, *E* = 130 kPa). In this case and others (Fig. 2d) we note that cells dividing in this manner will sometimes “re-fuse” after division before the next cell cycle. Further investigation is warranted to determine whether this behavior is indicative of a new class of checkpoint control programs that acts post-cytokinesis. Even considering potential corrective measures (re-fusion) that cells may employ, tri-daughter divisions (Fig. 2b & 2c), divisions resulting in unevenly sized daughter cells (Fig. 2c) and tetra-daughter divisions (Fig. 2d) are likely to result in increased susceptibility to abnormal chromosome segregation. As the HeLa cell line is a 3N+ karyotype, the amount of assembly of mitotic spindles from each centrosome is increased by at least 50% when compared to diploid cells. With extra centrosomes (N>2) and their associated mitotic spindles, metaphase and anaphase events are highly complex with respect to chromosome segregation to the poles even for bi-polar divisions. Even higher rates of missegregation is expected when cells divide in a multi-daughter fashion [5].

**Figure 3:**
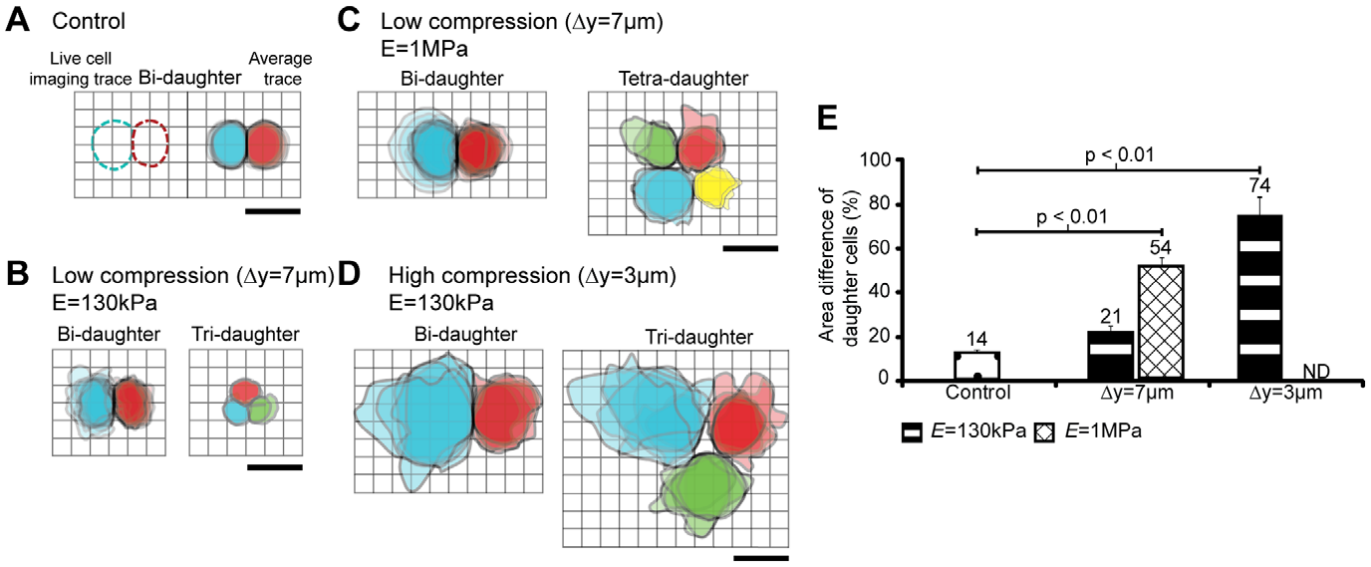
Daughter cell asymmetry and multi-daughter division increases with confinement and stiffness. Overlaid cell traces for control and all experimental conditions are shown. (a) Cell traces are analyzed 5 minutes post-telophase. Average control divisions are shown. (b,c) Low confinement average traces show increased size and asymmetry in stiffer environments. Notably, multi-daughter division type (tri- vs. tetra-daughter) depends on microenvironment mechanical properties. (d) In high confinement states, the daughter cells are more spread out and highly asymmetric when compared to both low compression and control cases. (e) Area difference between daughter cells in each test case demonstrating asymmetry differences. Error bars are SEM. All scale bars are 20 µm.

The degree of mechanical confinement affects the daughter cell size ratio in normal and multi-daughter cell divisions. Figure 3 illustrates the highly altered mitosis division shape and asymmetry under varied conditions of confinement. Upon the completion of telophase, cells in control conditions are ∼ 20 µm in width and highly uniform (Fig. 3a), whereas in the extreme case of high confinement the daughter cells span between 40–80 µm in width and are highly asymmetric (Fig. 3d). For intermediate conditions, both increased stiffness and confinement act to increase division asymmetry, as observed in the average traces (Fig. 3b, c). Quantification of daughter cell traces (Fig. 3e) demonstrates that the difference in area between daughter cells increases for all cases compared to control, where statistical significance (p<0.001) is observed between both low confinement high stiffness and high confinement low stiffness compared to the control.

Interestingly, a marked difference in type of multi-daughter division was observed when maintaining confinement, and changing only the substrate stiffness (Fig. 3a–d). Under low compression (Δy = 7 µm) when substrate stiffness is 130 KPa, multi-daughter divisions result primarily in three progeny (90%, Fig. 3b), however upon increasing substrate stiffness to 1 MPa the multi-daughter division mode shifts primarily to four progeny (85%, Fig. 3c). Assuming cell volume remains similar, the smaller cross-sectional cell area observed for softer 130 KPa substrates indicates that cells are able to apply significant force to deform the PDMS substrate and adopt a more rounded form. Note that a similar force is presumably applied by the cell to the stiffer 1 MPa substrate but this leads to less deformation. In both cases the force applied by the cell, and therefore the equal and opposite force applied by the substrate to the cell are similar, but the cell shape differs. Therefore, cell shape during mitosis may be a dominant factor in directing multi-daughter divisions (in which a more spherical shape is achievable for the softer substrates). A tendency towards tri- or tetra- daughter divisions may be due to spatial limits of chromosome assembly at the metaphase plane(s) in a confined shape, which is dependent on substrate stiffness. Alternatively, the location of mechanical loads may direct the geometry of these multi-daughter divisions, perhaps acting through cortical cues sensitive to environmental force magnitudes [23].

Along with possible effects of the uneven size of daughter cells and multi-daughter divisions on chromosomal segregation, alteration in cell shape with confinement may also pose difficulties in critical mitotic processes such as spindle assembly, signaling, and targeting processes during check point regulation and may be responsible for the increase in abnormal mitosis behavior. For example, signaling relying on either diffusion or active transport may proceed slower or be more error prone over the longer distances in an enlarged discoid shaped cell compared to a tight spherical cell. Additionally, different cytoskeletal filaments have a characteristic persistence length over which they are effective (i.e. able to transmit force between protein complexes) [24]. It is reasonable to postulate that by dramatically changing the shape of the cell from spherical to discoid, the lengths of force transmission (e.g. along microtubules from centrosome to kinetochore or from actin-based cortical cues to centrosomal microtubules [5], [23]) become anisotropic in varying directions of division, which may result in the underlying multi-polar and asymmetric divisions. Similarly, nonmuscle myosin II (NMM-II) distribution and contractility, necessary to create a uniform cleavage furrow during cytokinesis, may be affected by the non-spherical shape and anisotropic mechanical stress in these confined conditions. Fundamentally, these results suggest a role for adoption of a spherical shape during mitosis in maintaining bipolar division events.

**Figure 4:**
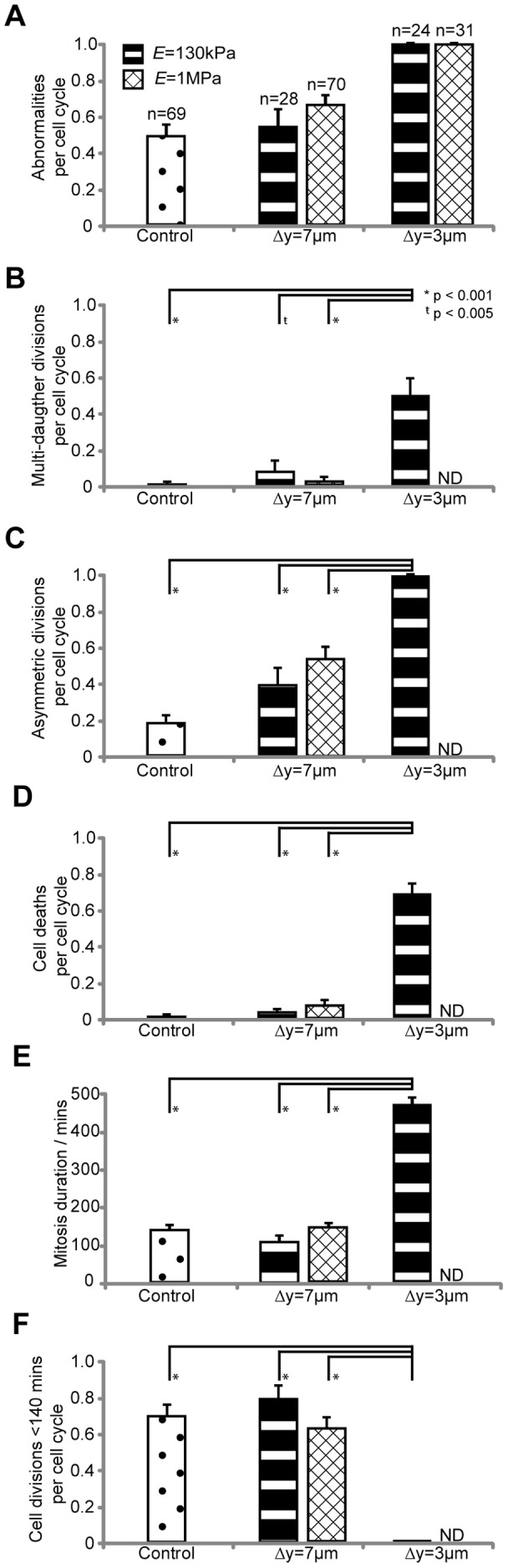
Quantitative effects of confinement on cell-cycle abnormalities. (a) Summary of experimental data in terms of abnormalities per cell cycle characterized by: multi-daughter divisions, asymmetric divisions, apoptotic divisions, and divisions lasting for more than 140 minutes. (b) Multi-daughter divisions per cell cycle characterized as divisions producing more than two daughter cells. Error bars are SEP. (c) Asymmetric divisions are characterized by daughter cells having a difference greater than 15% in area. Error bars are SEP. (d) Cell deaths per cell cycle are characterized by cells that cease activity and contract prior to completion of mitosis. Error bars are SEP. (e) Comparing the mitosis duration of the high confinement (Δy = 3 µm, *E* = 130 kPa) condition to control and both low confinement conditions. Error bars are SEM. (f) Summary of divisions in each case completing division within 140 minutes. For high confinement E = 1 MPa, no data (ND) was available as no completed mitosis events was observed. Error bars are SEP.

Mechanical confinement leads to statistically significant differences in the frequency of mitotic abnormalities. A summary of the abnormalities observed per cell cycle is shown in figure 4a. Here we define the total abnormalities per cell cycle as the combined frequency of multi-daughter divisions, divisions resulting in unevenly sized daughter cells, divisions resulting in cell death, and completed mitosis in greater than 140 minutes. Increasing both the geometric confinement of cells and substrate stiffness, leads to increasing frequencies of mitotic abnormalities (Fig. 4a). It should be noted that in the *E* = 1 MPa high confinement case no successful cell divisions were observed, but observable mitosis events (prophase, metaphase) were observed.

Increased frequencies of multi-daughter divisions are observed in highly confined environments. A summary of multi-daughter divisions per cell cycle is shown in figure 4b. In the unconfined control condition less than 1% of cell divisions generate more than two daughter cells. In contrast, under low confinement (Δy = 7 µm) slight increases in the frequency of multi-daughter divisions are observed, and in high confinement cases (Δy = 3 µm) a drastic increase to 50% of all divisions are observed to lead to multi-daughter generation. Further, cells which have undergone 1 to 3 divisions remain viable and produce progeny of their own. Video S6 shows a cell undergoing a 1 to 3 division, and subsequently one of the original daughter cells undergoing a second 1 to 3 division. This high frequency of multi-daughter divisions under confinement is unexpected, given that it is 17 times higher than the rate of multi-polar division events reported in standard 2D culture by Ganem et al. [4], and suggests a fundamental relationship where confinement and elasticity of the environment can control the geometry of the cell division plane(s).

Unevenly sized daughter cells, cell death rates, and mitosis duration increase with increasing confinement. Of the cells completing mitosis, the rate of unevenly sized (>15% difference) daughter cells produced per mitosis event significantly increased with both stiffness and confinement: up to 100% of all divisions under the highest confinement (Fig. 4c). In control conditions, mitosis resulting in cell death, possibly due to intact checkpoint failures, is observed in less than 1% of all division events. Cell death increases significantly to 4% and 8% at low confinement at *E* = 130 kPa and 1 MPa, respectively, and up to 70% under high confinement *E* = 130 kPa (Fig. 4d).

The duration of mitosis shown in figure 4e & 4f suggests increasing confinement is sufficient to increase mitotic duration. This corroborates previous observations by Kwon et al. [21] and Maresca et al. [17] that mitotic processes are dependent on correct orientation of, and force balance in, the cytoskeleton in order to achieve the proper signaling state to proceed through mitotic checkpoints in a timely manner.

**Figure 5:**
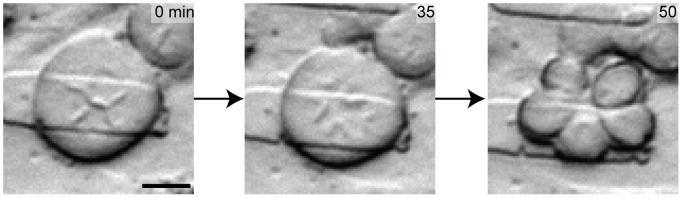
A penta-daughter cell division may lead to aneuploid daughter cells. As a qualitative example, this cell is in a partially confined state between 3–7 µm. Extreme confinement conditions observed here can lead to aberrant mitotic orientation resulting in highly asymmetric divisions. These microscopic images show the time lapse of a cell undergoing a single 1→5 successful mitosis. Scale bar: 20 µm.

A unique mitotic event resulting in five daughter cells under partial confinement was observed in which confinement was not complete such that the gap size was between 3–7 µm with *E* = 130 kPa (Fig. 5a). The cell in metaphase is 60 µm in diameter, 4 times wider than the 15 µm spherical cells undergoing mitosis during unconfined division. The metaphase lineup of chromosomes is highly abnormal as visual inspection suggests (Fig. 5a, left image) with at least 4 centrosome pole regions. Yet during anaphase (middle image), chromosome segregation seems to move toward 5 poles, leading to 5 daughter cells. Although the quantification of chromosome content is not available for these cells, it is reasonable to postulate that the chromosomes were incorrectly and unevenly segregated in this division, as with the other multi-daughter divisions (specifically to three daughter cells). In the case of the three daughter divisions, one replication cycle (S-phase), cannot be properly split into three without an extra half S-phase. In the case of the penta-daughter division, the same logic remains, however the mitotic planes appear to be even more complex. Although these penta-daughter division events are rare (observed twice over 500 single-cell observations), our single-cell analysis platform enables the capture of these rare atypical biological processes.

### Conclusion

To elucidate the effects of confined mechanical microenvironments on cell behavior and mitotic processes, the microfluidic platform described here successfully creates confined culture environments that may mimic aspects of *in vivo* tumor mechanical properties. The data presented shows high instances of abnormal multi-daughter division events induced simply by increasing the confinement of a cell (i.e. decreasing the available volume to divide in) and by increasing the substrate stiffness. Further, the frequencies at which these abnormalities occur is found at much higher rates than previously observed. The ability to induce these abnormal divisions to drastically higher rates (half of all divisions in some cases) suggests a fundamental interaction between the extracellular mechanical environment and the overall orientation and the multi-polarity of the cellular division axis.

The ability of this highly confined microenvironment to induce aberrant divisions, specifically multi-daughter divisions, is an intriguing finding in itself. Previously, work has been done to elucidate the biomolecular players involved in multi-centrosome divisions by Ganem et al. [4], however here we contribute by supporting the hypothesis that multi-daughter divisions may be due, at least in part, to abnormal mechanical interactions between the cell and the immediate environment.

The observations made and metrics analyzed here are purely phenotypic. While chromosome quantification is not presented here, these abnormal phenotypic divisions strongly suggest aberrations in chromosome segregation, and should be investigated molecularly. These results provide strong initial evidence for the dependence of the mitotic geometry and polarity on the extracellular mechanical environment, and warrants dedicated, in-depth molecular characterization of the phenomena.

## Materials and Methods

### Microdevice Design

To simulate aspects of a compliant confinement microenvironment we designed a perfusable microfluidic device with a compressible assay chamber (4 mm×6 mm) (Fig. 1). This setup allows for ease of observation while simulating a pseudo-3D environment. The device’s low fluidic resistance allows uniform high density cell seeding and low shear stress during perfusion. The device operates under continuous perfusion with a total chamber volume exchange every two minutes to maintain the cells during assays. The array of 20 µm×80 µm posts spaced 40 µm apart within the chamber provides control of the confinement height, structural support to resist sagging, and a pseudo-3D confined environment. In contrast to 3D culture in hydrogel systems our system provides precise control of the confinement volume, active uniform delivery of nutrients to each cell, and a single imaging plane.

### Microfluidic Chip Fabrication

The master molds for the mechanical micro-confinement culture device were fabricated using negative photoresist (SU-5, Microchem Corporation). The base layer was spun on at 950 RPM to yield a 15 µm channel height, and the post heights (Δy) were 7 µm and 3 µm, corresponding to 1750 RPM and 3300 RPM, respectively. Poly(dimethylsiloxane) (PDMS) was prepared with varying (v/v) ratios of crosslinker to base (1∶10 for *E* = 1 MPa and 1∶20 for *E* = 130 kPa) [25]. The elastomer devices were cured at 65°C overnight, then cut from the mold, punched, and bonded to clean glass slides (Fisher Scientific) after treatment of both the glass and PDMS with Oxygen Plasma (0.5 Torr, 35 W) for 20 seconds.

### Cell Culture & Experimental Setup

Human cervical carcinoma (HeLa) cells were purchased from American Type Culture Collection (ATCC, Manassas, VA, catalog CCL-2). Cells were maintained in high glucose Dulbecco’s modified Eagle’s medium (DMEM), 100 µg mL^−1^ penicillin, 100 µg mL^−1^ streptomycin, and 10% fetal bovine serum (Gibco). Cell synchronization was performed by a two day serum starve followed by a 2 mM double thymidine block [26]. Prior to the assay, the device was primed and flushed with ethanol at 20 µL min^−1^ for 15 minutes, followed by a PBS flush at 20 µL min^−1^ for 15 minutes. The device was then coated with bovine fibronectin (Invitrogen) 50 µg mL^−1^ adsorbed nonspecifically for 90 minutes at room temperature. Cells were loaded as a suspension of 1 million cells mL^−1^ to achieve a final in-device density of approximately 100 cells per 600 µm^2^ in the confinement assay area (Fig. 1a). Cells were then placed in a microscope incubator for 20 minutes to allow for attachment.

For confinement assays, the device was compressed via a calibrated weight. To determine the proper mass to use for each confinement and stiffness case, a separate device, not containing cells, perfused with blue dye was used to determine when the posts came in contact with the glass substrate (i.e. the posts became optically clear). This process is graphically represented in figure 1b. The mass of calibration weights (80 g–100 g) were also confirmed by streak imaging of 2.2 µm beads which allowed tracking of compression progress (Fig. S1). Once the beads were no longer transiting under the posts, an effective calibration weight was arrived at. The heights of the devices were kept approximately constant by pouring an equal volume of PDMS:crosslinker each time a new device was molded from the master to achieve a bulk height of approximately 5 mm. Upon confinement, cells are subjected to a confinement force that is equal to the force exerted by the cell onto the substrate. This confinement force is dependent on the confinement height but not the substrate elasticity.

The perfusion of supplemented DMEM media began at one half of a volume exchange of the chamber per minute (750 nL min^−1^). Live-cell imaging was performed in a microscope incubator (temperature and CO_2_ controlled) with an inverted microscope (Nikon Eclipse TI). Images were captured with a Coolsnap HQ2 camera on the Nikon Advance Research software (Nikon) every five minutes with experiments lasting up to six days.

The mechanical microenvironment of the cell is defined by the spatial confinement volume and elastic modulus of the substrate. As the PDMS membrane is approximately 5 mm thick for each device, a large damping of the applied force to lower the chamber is expected such that the total effective force at the chamber cell interface is dependent on the elasticity of the polymer matrix with minimal dependence on the force used to lower the chamber. In the case of a thin PDMS membrane the applied force would have a significant impact on the total effective force on the cell, however this is not the case in this system. In figure S2, we further investigate this by examining whether the load used to compress the cells affects the cell’s ability to deform the PDMS substrate. The numerical simulations do not show a difference between load and no-load conditions.

### Analysis

Before extracting quantitative data on cell morphology the completeness of confinement was confirmed for each cell video by observing that no cells in the FOV transited under posts. In this way we ensured an expected and repeatable height of confinement was achieved. ImageJ was used to perform analysis on the images obtained experimentally. Specifically, the area function was used to calculate the areas of daughter cells after cytokinesis by manually outlining each daughter cell, after a complete cleavage furrow and separation was observed. Each mitosis event was manually identified and characterized. The onset of mitosis was defined as the point at which the cell lost its spindled morphology and became completely rounded. It should be noted that in highly confined cases, mitosis onset was identified by the appearance of a well defined circular cell edge, as most of the cells did not adopt the classical spindled morphology under high confinement.

For statistical analysis, the population number (n) used corresponds to the total number of events observed in a single category of abnormality (e.g. multi-daughter divisions, uneven divisions, etc). These sample sizes were created by repeating each experimental condition two times, meaning two separate devices. The standard errors of the ‘per division’ metrics were found using the standard error of the estimate of a proportion. Confidence intervals between the mitotic duration times were performed with the Welch t-test, as the distributions did not have the same standard deviations. The confidence intervals in comparing proportions, such as abnormalities per cell cycle, etc were calculated using the z statistic for proportions.

Solid mechanics numerical simulation was performed using COMSOL v4.2 (Los Angeles, CA, USA).

## Supporting Information

Figure S1 **Device compression as qualified by streak imaging of 2.2 µm beads.** Uncompressed 7 µm device (left), fully compressed 7 µm device (right). Scale bars 50 µm.
(TIF)

Figure S2 **COMSOL simulation of effective force at the cell interface.** Numerical simulations comparing a 1 µm substrate deformation and the resulting stresses between no-load and a calibrated 15 kPa load with a 5 mm bulk PDMS layer. A) Simulation setup for no load and 15 kPA load. B) Interfacial stresses at the cell interface and substrate. Scale bars 10 µm.
(TIF)

Video S1 **7** **µm height 130** **kPa condition in 72 hour time lapse.**
(WMV)

Video S2 **Tri-daughter cytokinesis.** Time lapsed 38 hours.
(AVI)

Video S3 **Asymmetric division.** Time lapsed 4 hours.
(AVI)

Video S4 **Tetra-daughter cytokinesis.** Time lapsed 4 hours.
(AVI)

Video S5 **Apoptotic division.** Time lapsed 2 hours.
(AVI)

Video S6 **Sequential tri-daughter cytokinesis.** Time lapsed 40 hours.
(AVI)

## References

[1] Duncan AW, Taylor MH, Hickey RD, Hanlon Newell AE, Lenzi ML (2010). The ploidy conveyor of mature hepatocytes as a source of genetic variation.. Nature.

[2] Erenpreisa J, Cragg MS (2010). MOS, aneuploidy and the ploidy cycle of cancer cells.. Oncogene.

[3] Vitale I, Senovilla L, Jemaà M, Michaud M, Galluzzi L (2010). Multipolar mitosis of tetraploid cells: inhibition by p53 and dependency on Mos.. EMBO J.

[4] Ganem NJ, Godinho SA, Pellman D (2009). A Mechanism Linking Extra Centrosomes to Chromosomal Instability.. Nature.

[5] Kops GJPL, Weaver BAA, Cleveland DW (2005). On the road to cancer: aneuploidy and the mitotic checkpoint.. Nat Rev Cancer.

[6] Howard A, Pelc SR (1956). A difference between spermatogonia and somatic tissues of mice in the incorporation of [8–14C]-adenine into deoxyribonucleic acid.. Exp Cell Res.

[7] Shyy JY-J, Chien S (2002). Role of integrins in endothelial mechanosensing of shear stress.. Circ Res.

[8] Bershadsky AD, Balaban NQ, Geiger B (2003). Adhesion-dependent cell mechanosensitivity.. Annu Rev Cell Dev Biol.

[9] Ingber DE (2008). Can cancer be reversed by engineering the tumor microenvironment?. Semin Cancer Biol.

[10] Lelièvre SA, Weaver VM, Nickerson JA, Larabell CA, Bhaumik A (1998). Tissue phenotype depends on reciprocal interactions between the extracellular matrix and the structural organization of the nucleus.. Proc Natl Acad Sci U S A.

[11] Kilian KA, Bugarija B, Lahn BT, Mrksich M (2010). Geometric cues for directing the differentiation of mesenchymal stem cells.. Proc Natl Acad Sci USA.

[12] Kumar S, Weaver VM (2009). Mechanics, malignancy, and metastasis: The force journey of a tumor cell. Cancer Metastasis Rev 28: 113–127.. doi.

[13] Alexander NR, Branch KM, Parekh A, Clark ES, Iwueke IC (2008). Extracellular matrix rigidity promotes invadopodia activity.. Curr Biol.

[14] Assoian RK, Klein EA (2008). Growth control by intracellular tension and extracellular stiffness.. Trends Cell Biol.

[15] Théry M, Bornens M (2008). Get round and stiff for mitosis.. HFSP J.

[16] Musacchio A, Salmon ED (2007). The spindle-assembly checkpoint in space and time.. Nat Rev Mol Cell Biol.

[17] Maresca TJ, Salmon ED (2010). Welcome to a new kind of tension: translating kinetochore mechanics into a wait-anaphase signal.. J Cell Sci.

[18] Peters J-M (2006). The anaphase promoting complex/cyclosome: a machine designed to destroy.. Nat Rev Mol Cell Biol.

[19] Théry M, Racine V, Pépin A, Piel M, Chen Y (2005). The extracellular matrix guides the orientation of the cell division axis.. Nat Cell Biol.

[20] Vilmos P, Jankovics F, Szathmári M, Lukácsovich T, Henn L (2009). Live imaging reveals that the Drosophila actin-binding ERM protein, moesin, co-localizes with the mitotic spindle.. Eur J Cell Biol.

[21] Kwon M, Godinho SA, Chandhok NS, Ganem NJ, Azioune A (2008). Mechanisms to suppress multipolar divisions in cancer cells with extra centrosomes.. Genes Dev.

[22] Park AYJ, Shen T-L, Chien S, Guan J-L (2009). Role of focal adhesion kinase Ser-732 phosphorylation in centrosome function during mitosis.. J Biol Chem.

[23] Théry M, Jiménez-Dalmaroni A, Racine V, Bornens M, Jülicher F (2007). Experimental and theoretical study of mitotic spindle orientation.. Nature.

[24] Gittes F, Mickey B, Nettleton J, Howard J (1993). Flexural rigidity of microtubules and actin filaments measured from thermal fluctuations in shape.. J Cell Biol.

[25] Ochsner M, Dusseiller MR, Grandin HM, Luna-Morris S, Textor M (2007). Micro-well arrays for 3D shape control and high resolution analysis of single cells.. Lab Chip.

[26] Stein GS, Borun TW (1972). The synthesis of acidic chromosomal proteins during the cell cycle of HeLa S-3 cells. I. The accelerated accumulation of acidic residual nuclear protein before the initiation of DNA replication.. J Cell Biol.

