# Supplementary figures and images for: Increased Asymmetric and Multi-Daughter Cell Division in Mechanically Confined Microenvironments
Source: PLoS One. 2012 Jun 25;7(6):e38986. doi: 10.1371/journal.pone.0038986 (PMC3382600; doi:10.1371/journal.pone.0038986)

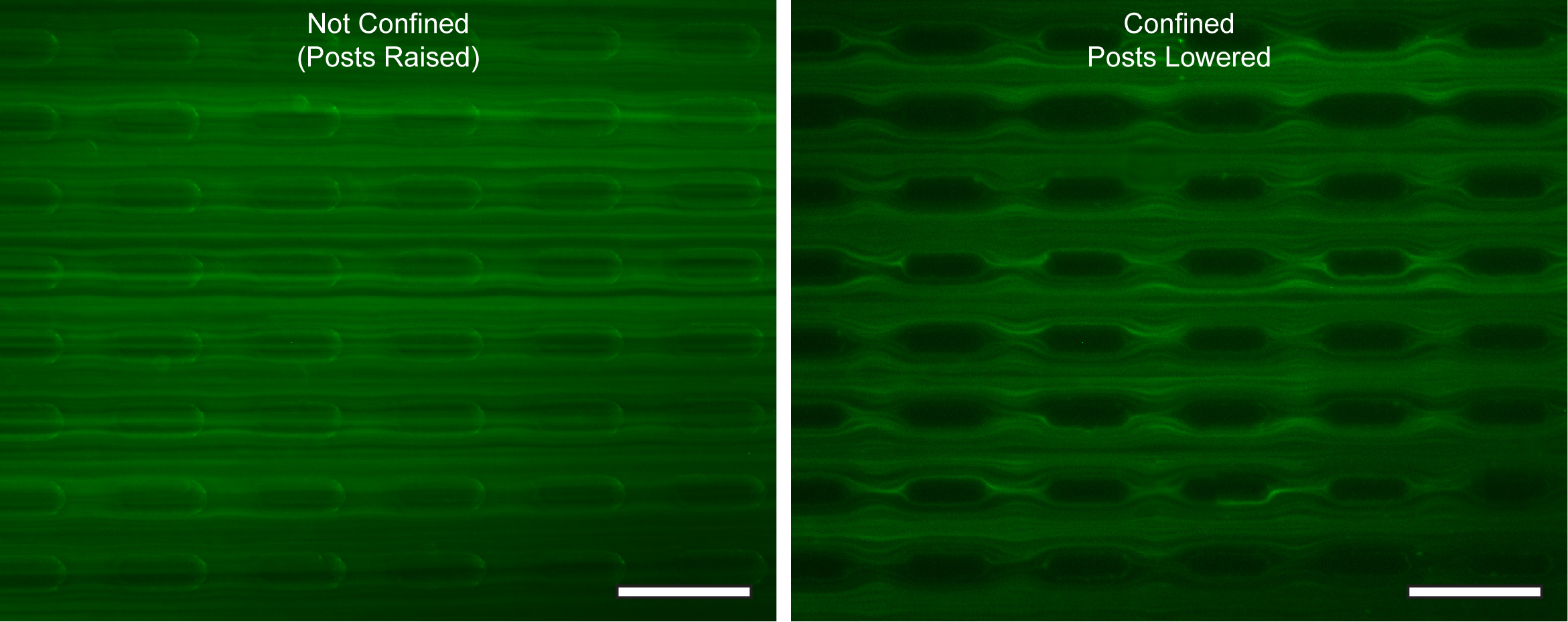

Supplement: Figure S1 — Device compression as qualified by streak imaging of 2.2 µm beads. Uncompressed 7 µm device (left), fully compressed 7 µm device (right). Scale bars 50 µm. (TIF) [file pone.0038986.s001.tif]

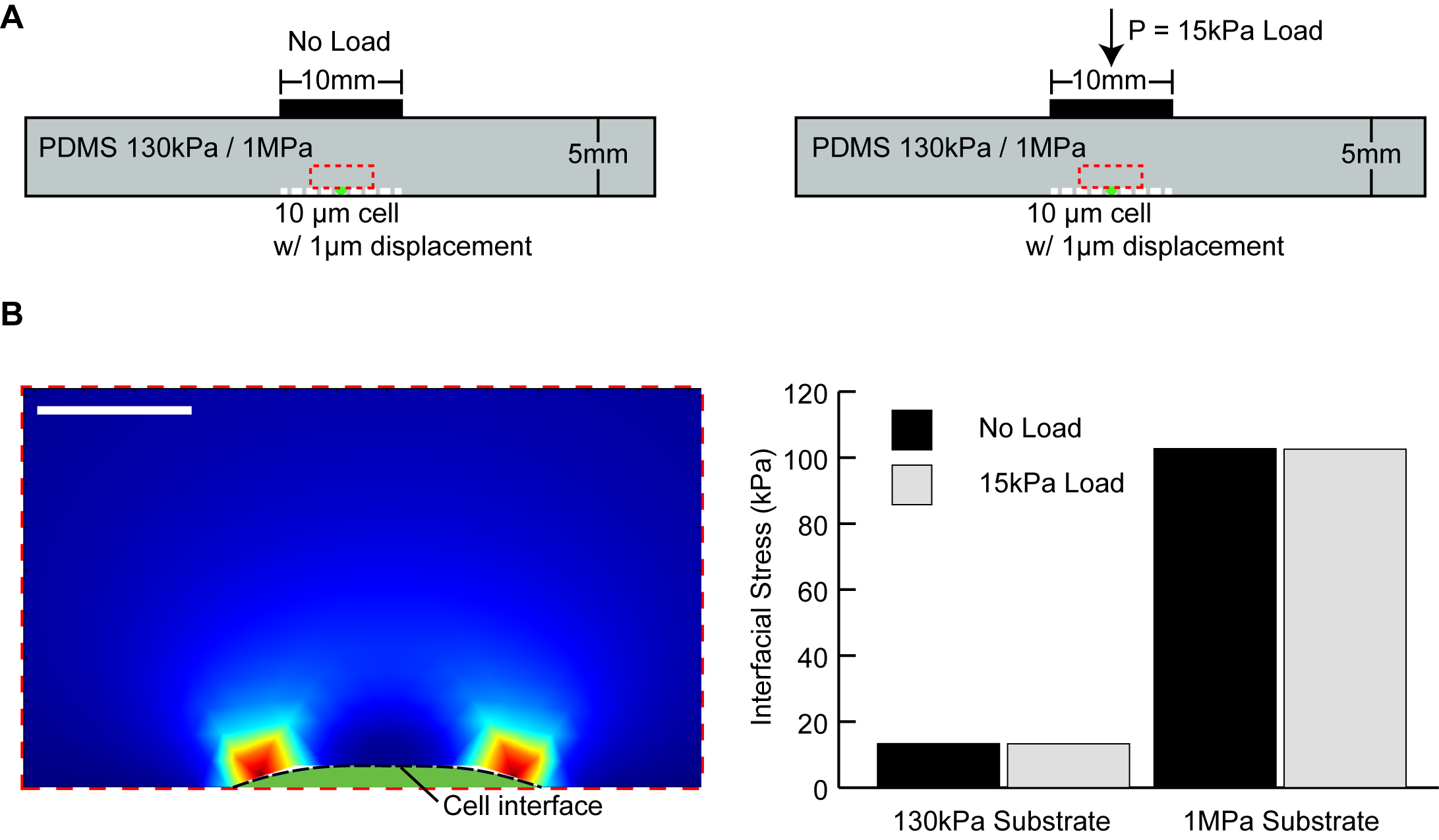

Supplement: Figure S2 — COMSOL simulation of effective force at the cell interface. Numerical simulations comparing a 1 µm substrate deformation and the resulting stresses between no-load and a calibrated 15 kPa load with a 5 mm bulk PDMS layer. A) Simulation setup for no load and 15 kPA load. B) Interfacial stresses at the cell interface and substrate. Scale bars 10 µm. (TIF) [file pone.0038986.s002.tif]
